# Photobiomodulation in Age-Related Macular Degeneration: A Mitochondrial Bioenergetic Framework and Translational Perspective

**DOI:** 10.3390/life16071098

**Published:** 2026-06-30

**Authors:** Thomas Desmettre, Serge Mordon

**Affiliations:** 1 Department of Ophthalmology, University of Kansas School of Medicine, Prairie Village, KS 66208, USA; 2 Centre de Rétine Médicale, 187 Rue de Menin, 59520 Marquette-Lez-Lille, France; 3 Hemerion Therapeutics, 37 Rue Denis Papin, 59650 Villeneuve-d’Ascq, France; srm@hemerion.com

**Keywords:** age-related macular degeneration, photobiomodulation, mitochondrion, retinal pigment epithelium, bioenergetics

## Abstract

**Background/Objectives**: Age-related macular degeneration (AMD) is a leading cause of irreversible visual impairment in aging populations. While effective treatments exist for neovascular AMD and, more recently, geographic atrophy, no widely accepted therapy prevents disease progression in earlier stages. Photobiomodulation (PBM) has emerged as a potential approach to modulate retinal metabolism. This review examines the biological mechanisms proposed to explain PBM effects and explores how mitochondrial physiology may support a bioenergetic framework linking these mechanisms to retinal function in AMD. **Methods**: We conducted a targeted review of experimental and clinical literature addressing photobiomodulation, mitochondrial function, and retinal metabolism in AMD. **Results**: Experimental studies indicate that PBM may influence mitochondrial activity through multiple mechanisms, including modulation of cytochrome c oxidase, nitric oxide photodissociation, and redox signaling pathways. However, the diversity of mechanisms and variability of clinical outcomes suggest that the biological basis of PBM remains incompletely understood. Emerging insights from mitochondrial physiology, including the debated concept of local thermal microenvironments, support interpreting PBM effects as modulation of mitochondrial bioenergetics rather than isolated molecular pathways. **Conclusions**: We propose a conceptual framework in which PBM responses are governed by the interaction between a dose-dependent bioenergetic window and the mitochondrial functional reserve of retinal cells. Within this model, therapeutic effects are most likely when PBM is delivered within an optimal range of stimulation and in retinal tissues where mitochondrial dysfunction remains partially reversible. This framework may help explain variability across studies and support more rational use of PBM in early and intermediate AMD.

## 1. Introduction

Age-related macular degeneration (AMD) is a leading cause of irreversible visual impairment in elderly populations worldwide and represents a major public health concern in aging societies [1,2]. While effective treatments exist for neovascular AMD [3] and, more recently, for geographic atrophy [4], there is currently no widely accepted therapy capable of preventing disease progression during the earlier stages of the disease. Increasing attention has therefore been directed toward the identification and management of early disease phenotypes [5], including early and intermediate AMD [6], as well as subretinal drusenoid deposits (SDDs) [7,8], which may reflect distinct pathogenic pathways and risks of progression.

Growing evidence indicates that mitochondrial dysfunction plays a central role in AMD pathogenesis. Photoreceptors require substantial amounts of ATP to sustain phototransduction and maintain ionic gradients, making retinal metabolism one of the most energy-demanding processes in the human body [9]. In parallel, retinal pigment epithelium (RPE) cells rely on mitochondrial oxidative phosphorylation to support essential functions such as photoreceptor outer segment phagocytosis and retinoid recycling within the visual cycle [10]. These metabolic constraints render the RPE–photoreceptor complex particularly vulnerable to age-related mitochondrial alterations [11].

Among the therapeutic approaches proposed for these earlier stages, photobiomodulation (PBM) has attracted increasing interest [12,13,14]. PBM uses low-intensity visible or near-infrared light to influence cellular processes and has been investigated across neurological and ophthalmic conditions [15,16]. Experimental and early clinical studies suggest that PBM may influence retinal metabolism and cellular homeostasis. However, despite a growing body of literature, recent reviews remain cautious regarding the magnitude and consistency of clinical benefits reported in AMD [17,18].

Clinical studies of PBM in AMD have yielded heterogeneous results, which appear to depend in part on disease stage and baseline retinal status. While functional improvements have been reported in early and intermediate AMD [14], outcomes in more advanced stages, such as geographic atrophy, have been more limited and inconsistent [17,18]. These observations suggest that biological responsiveness to PBM may vary according to disease stage and the underlying metabolic condition of retinal tissues.

As summarized in Table 1, most published clinical studies have reported improvements in visual function, including best-corrected visual acuity, low-luminance visual acuity, contrast sensitivity, or retinal sensitivity. However, evidence supporting true disease modification remains less robust, as structural endpoints such as drusen regression, geographic atrophy progression, or prevention of neovascular conversion have been evaluated less consistently.

An additional observation emerging from the clinical literature is the progressive focus on earlier stages of AMD. Most recent studies have preferentially enrolled patients with early or intermediate disease, consistent with the hypothesis that preserved mitochondrial functional reserve may be required for optimal responsiveness to PBM. Similarly, the currently available evidence is concentrated in patients with residual retinal structure and function, whereas data remain limited for advanced atrophic stages, where irreversible cellular loss may constrain the biological response to treatment.

While clinical studies provide encouraging signals, the biological mechanisms underlying PBM remain incompletely understood. A wide range of biological mechanisms has been proposed to explain the effects of PBM (Table 2), including modulation of mitochondrial respiratory activity through cytochrome c oxidase [15], photodissociation of nitric oxide from the respiratory chain [16], activation of redox-sensitive signaling pathways [19], and modulation of inflammatory responses [16]. While these mechanisms are often presented as complementary, they remain difficult to integrate into a unified framework linking light–tissue interactions to the variability of metabolic and clinical responses observed in AMD.

Because many of these mechanisms converge on mitochondrial respiration, increasing attention has been directed toward the interaction between light irradiation and mitochondrial physiology. Recent studies have suggested that actively respiring mitochondria may operate within specific thermal microenvironments that could influence the efficiency of oxidative phosphorylation [20,21]. Although the magnitude and physiological relevance of such mitochondrial temperature gradients remain debated [22,23], these observations highlight the need to better characterize the physical and bioenergetic context in which PBM acts.

**Table 1:** Summary of major clinical studies evaluating photobiomodulation in age-related macular degeneration. The table compares the principal clinical studies of photobiomodulation (PBM) in AMD according to publication year, study design, patient population, disease stage, treatment protocol, follow-up duration, primary endpoints, reported outcomes, and corresponding references.

Study	Design	Patients	AMD Stage	PBM Protocol	Follow-Up	Primary Endpoint	Key Outcomes	References
TORPA I	Pilot study	18 eyes	Dry AMD	590, 670, and 790 nm LED	12 mo	VA, contrast sensitivity	Functional improvement	Merry et al., 2012 [24]
TORPA II	Pilot study	24 patients (42 eyes)	Dry AMD	590, 670, and 790 nm LED	3 mo	Visual function	Functional improvement	Merry et al., 2017 [25]
LIGHTSITE II	Randomized sham-controlled	44 patients (53 eyes)	Intermediate AMD	590, 660, and 850 nm; 9 sessions/cycle	10 mo	BCVA	Functional improvement with anatomical signals	Burton et al., 2023 [13]
LIGHTSITE III	Randomized sham-controlled	100 patients (148 eyes)	Intermediate AMD	590, 660, and 850 nm; repeated treatment cycles	13 mo	BCVA	Sustained functional benefit; exploratory structural effects	Boyer et al., 2024 [12]
LIGHTSITE III	Randomized sham-controlled	100 patients (148 eyes)	Intermediate AMD	590, 660, and 850 nm; repeated treatment cycles	24 mo	BCVA	Sustained visual benefit; possible signal for reduced GA incidence	Jaffe et al., 2026 [14]
PBM4AMD	Prospective observational	38 patients	Early/Intermediate AMD	590, 660, and 850 nm (Valeda^®^ protocol)	24 weeks	BCVA, LLVA	Short-term functional improvement; structural effects uncertain	Nassisi et al., 2024 [26]

**Table 2:** Proposed biological mechanisms underlying photobiomodulation and their level of experimental support. Evidence levels were assigned qualitatively for this review. Strong evidence indicates support from multiple independent studies, moderate evidence indicates partial or indirect support, and theoretical evidence indicates biologically plausible mechanisms that remain to be experimentally validated.

Mechanism	Evidence Level	References
Cytochrome c oxidase (CCO) photoactivation and nitric oxide (NO) photodissociation	Strong experimental	Hamblin 2018 [27]
Increased mitochondrial respiration and ATP production	Moderate experimental	Fuma 2015 [28]
Redox signaling pathways (e.g., Nrf2, PGC-1α activation)	Moderate experimental	Ponnusamy [29]
Modulation of inflammatory pathways	Limited/emerging experimental	Chung 2012 [15]
Mitochondrial thermal microenvironment hypothesis	Theoretical/debated	Chrétien 2018 [20]
Integration of PBM effects through mitochondrial bioenergetic modulation (dose-dependent window and mitochondrial functional reserve)	Conceptual/integrative	This conceptual framework

In this review, we examine photobiomodulation in the context of retinal physiology and AMD, with a particular focus on mitochondrial bioenergetics. Building on experimental and clinical observations, we propose a conceptual framework in which PBM effects are interpreted through two interacting dimensions: a dose-dependent bioenergetic response and a stage-dependent mitochondrial functional reserve. This framework aims to provide a mechanistic basis for the variability observed across studies and to better define the conditions under which PBM may exert clinically meaningful effects.

## 2. Methods

This review was conducted as a targeted narrative review. Literature searches were performed in PubMed/MEDLINE and Web of Science through January 2026 using combinations of the terms “photobiomodulation”, “low-level light therapy”, “age-related macular degeneration”, “mitochondria”, “oxidative phosphorylation”, “retinal pigment epithelium”, and “retina”. Additional relevant publications were identified through manual review of reference lists from key articles.

Priority was given to peer-reviewed original studies, clinical trials, systematic reviews, and landmark mechanistic investigations relevant to PBM, retinal bioenergetics, and AMD pathophysiology. Studies not directly related to retinal disease or mitochondrial mechanisms were excluded. References were selected according to their relevance to the interaction between light irradiation, mitochondrial function, and retinal metabolic homeostasis.

As a targeted narrative review, this work was not designed to provide a systematic or exhaustive assessment of all published literature on PBM in AMD. Rather, studies were selected to support the development of a mechanistic and translational framework integrating current experimental and clinical evidence.

In addition to summarizing published evidence, this review integrates experimental, translational, and clinical findings to develop a unified conceptual framework linking PBM-mediated modulation of mitochondrial bioenergetics with disease stage-dependent responses in AMD.

## 3. Mitochondrial Bioenergetics and Metabolic Vulnerability in the Retina

Retinal tissue is among the most metabolically active in the human body and exhibits one of the highest oxygen consumption rates of any tissue [30]. Photoreceptors continuously consume large amounts of ATP to sustain phototransduction and maintain ionic gradients across their plasma membranes, representing a major energetic cost in retinal physiology [9]. In parallel, RPE cells perform several energy-demanding processes, including phagocytosis of photoreceptor outer segments and retinoid recycling within the visual cycle [10]. As a result, the photoreceptor–RPE complex operates under sustained metabolic demand and relies heavily on mitochondrial oxidative phosphorylation to maintain retinal homeostasis.

**Figure 1:**
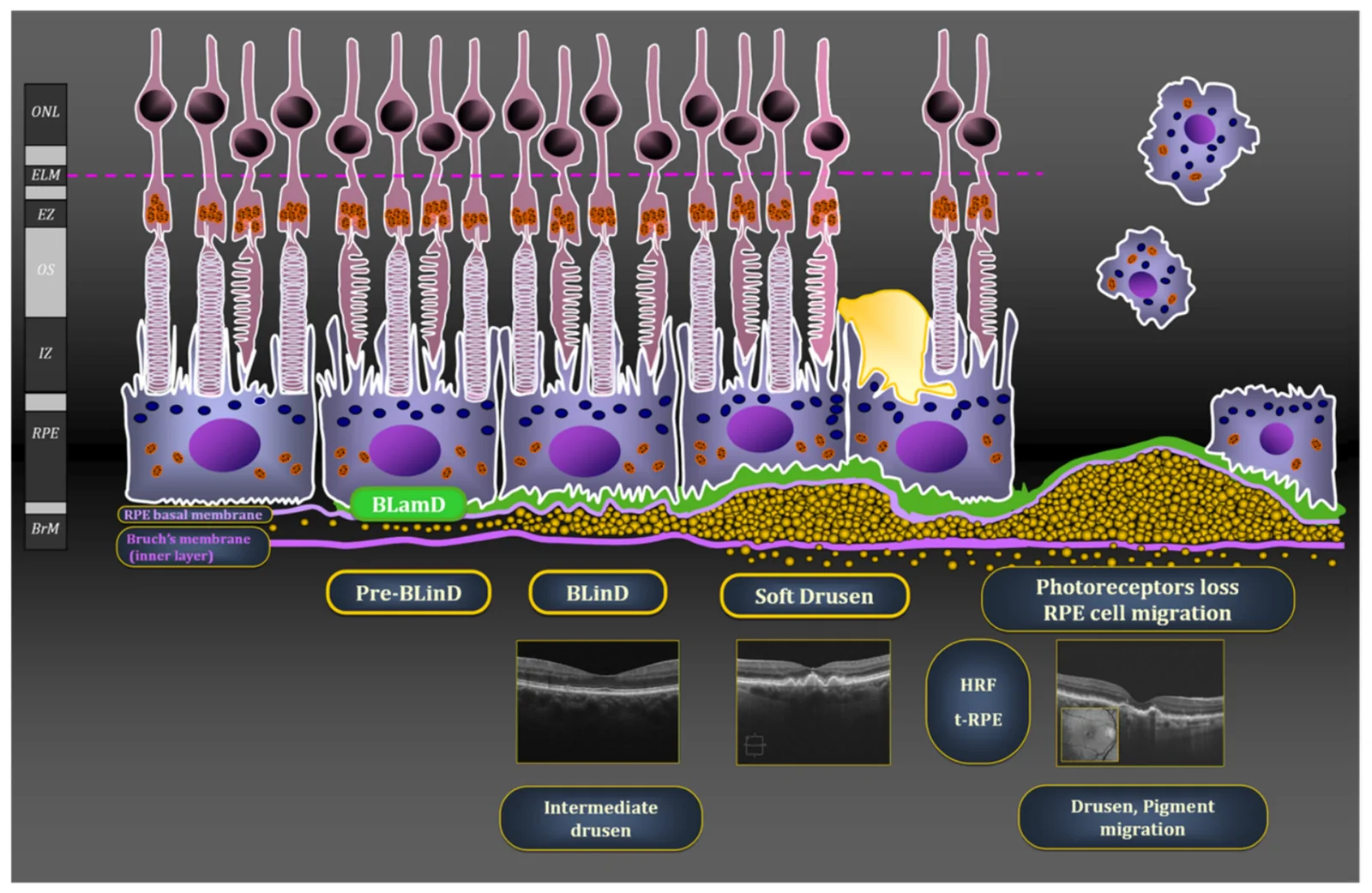
Outer retinal organization and structural features in early and intermediate AMD. Schematic representation of the photoreceptor–retinal pigment epithelium (RPE) complex and the principal structural biomarkers associated with early AMD progression. Photoreceptor inner segments contain a high density of mitochondria within the ellipsoid region, corresponding to the ellipsoid zone (EZ) on optical coherence tomography (OCT). The diagram illustrates the localization of conventional drusen, subretinal drusenoid deposits (SDDs), hyperreflective foci (HRF), transdifferentiated RPE cells (tRPE), and basal deposits (BLamD, pre-BLinD, and BLinD). Conceptual illustration inspired by histological and imaging studies of the outer retina and early AMD deposits [2,37,38]. Abbreviations: RPE, retinal pigment epithelium; OCT, optical coherence tomography; EZ, ellipsoid zone; SDDs, subretinal drusenoid deposits; HRF, hyperreflective foci; tRPE, transdifferentiated retinal pigment epithelium cells; BLamD, basal laminar deposits; pre-BLinD, pre-basal linear deposits; BLinD, basal linear deposits.

Photoreceptor inner segments are densely packed with mitochondria, particularly within the ellipsoid region. This high mitochondrial density corresponds to the hyperreflective “ellipsoid zone” observed on optical coherence tomography (OCT) (Figure 1), which provides an indirect indicator of photoreceptor bioenergetic integrity [31,32].

Growing evidence indicates that mitochondrial dysfunction plays a central role in the pathogenesis of AMD. Structural alterations of the mitochondrial network, increased mitochondrial DNA damage, and impaired respiratory chain activity have been described in aging RPE cells and AMD tissues [11,33,34,35,36]. These changes are associated with reduced oxidative phosphorylation efficiency, increased oxidative stress, and diminished cellular resilience, supporting a direct contribution of mitochondrial dysfunction to retinal metabolic vulnerability.

Beyond structural alterations, age-related impairment of mitochondrial dynamics and quality-control pathways further contributes to retinal degeneration [11]. Dysregulation of mitochondrial biogenesis, mitophagy, and redox homeostasis may progressively reduce the metabolic efficiency of the RPE–photoreceptor unit. Because photoreceptors operate close to their metabolic limits, even modest reductions in mitochondrial efficiency may have significant functional consequences. In this context, the concept of a limited mitochondrial functional reserve provides a useful framework to interpret the progressive loss of cellular resilience observed in aging and disease.

**Figure 2:**
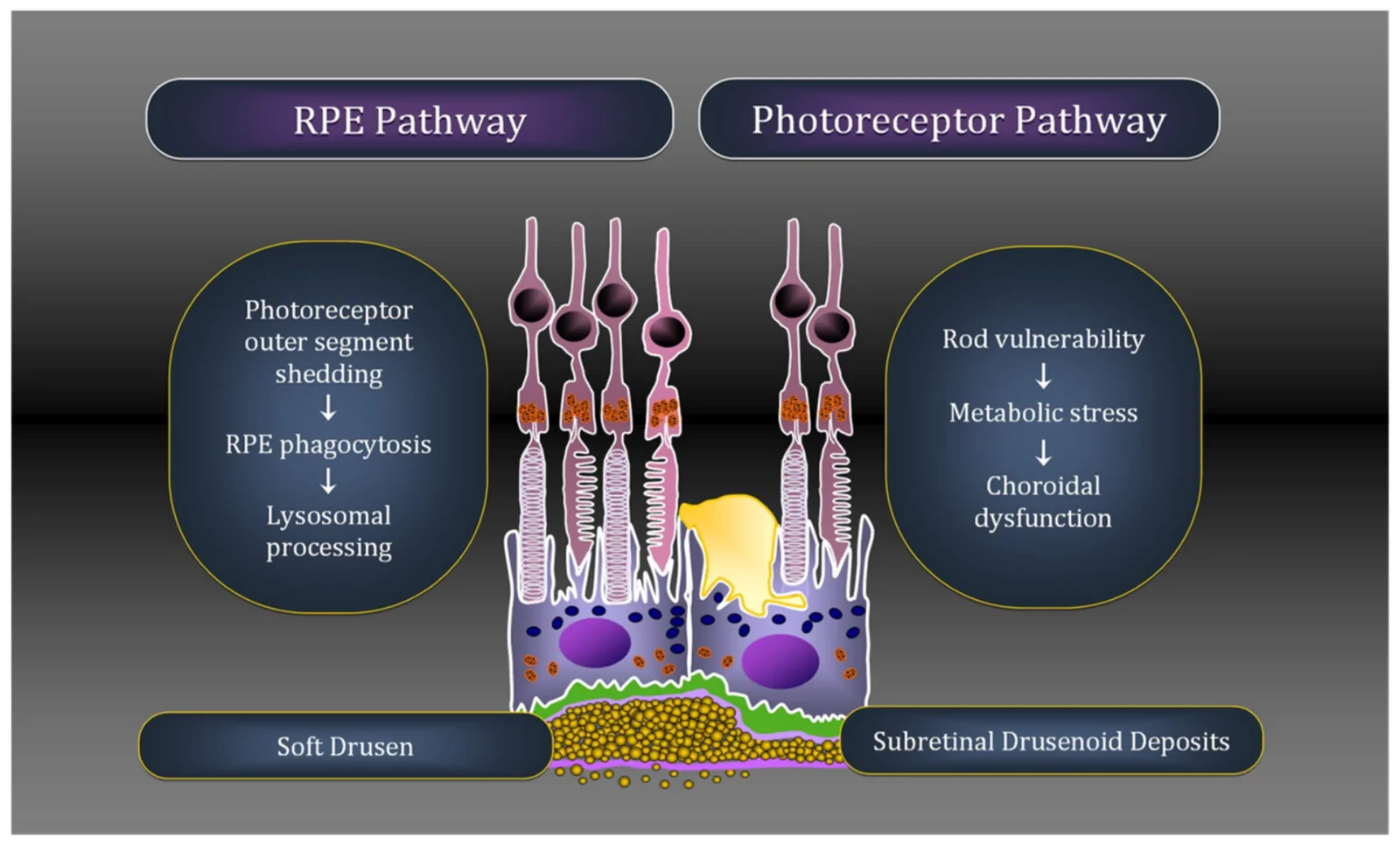
Distinct pathogenic pathways for drusen and subretinal drusenoid deposits. Schematic representation of two major pathogenic pathways in early AMD. Conventional drusen are primarily associated with retinal pigment epithelium (RPE) dysfunction, whereas subretinal drusenoid deposits (SDDs) are linked to photoreceptor and choroidal dysfunction, reflecting distinct metabolic vulnerabilities within the outer retina [7,8].

Recent observations also suggest that distinct pathogenic pathways contribute to early AMD. Conventional drusen are generally thought to arise from alterations in lipid handling and lysosomal processing within the RPE following the daily phagocytosis of photoreceptor outer segments [37,38]. In contrast, subretinal drusenoid deposits (SDDs) appear to be associated with dysfunction of photoreceptors and the choroidal circulation, particularly in rod-rich regions. These deposit types frequently coexist but may reflect distinct metabolic vulnerabilities within the outer retina (Figure 2) [7,8].

## 4. Photobiomodulation as a Modulator of Mitochondrial Bioenergetics

Photobiomodulation (PBM), historically referred to as low-level light therapy, uses visible red or near-infrared light delivered at low irradiance to influence cellular metabolism [16]. Experimental studies over the past two decades have shown that PBM can modulate mitochondrial function and cellular bioenergetics across a range of biological systems [15]. These findings have led to increasing interest in PBM as a potential approach to influence retinal metabolism in diseases associated with mitochondrial dysfunction, including AMD.

Experimental work from Jeffery and colleagues provides evidence that long-wavelength light exposure can improve retinal function through modulation of mitochondrial activity. Studies in animal models and human subjects have shown that exposure to 670 nm light can enhance photoreceptor performance, improve mitochondrial efficiency, and modulate inflammatory markers, particularly in aging retinas [39,40]. These observations support the biological plausibility that PBM-induced modulation of mitochondrial bioenergetics may translate into measurable functional effects.

The most widely proposed intracellular target of PBM is cytochrome c oxidase (complex IV) of the mitochondrial respiratory chain. Action spectrum studies suggest that several wavelengths in the red and near-infrared range correspond to absorption peaks of this enzyme, supporting its role as a primary photoacceptor [15]. Photon absorption by cytochrome c oxidase has been proposed to influence electron transport within the respiratory chain, thereby modulating mitochondrial membrane potential and ATP production [16]. In addition, nitric oxide-mediated reversible inhibition of cytochrome c oxidase has been proposed as a complementary mechanism, whereby light-induced photodissociation of nitric oxide restores electron flow and mitochondrial respiration [16]. These mitochondrial effects are associated with transient changes in reactive oxygen species production and activation of downstream signaling pathways involved in cellular stress responses [15].

Although cytochrome c oxidase has emerged as the principal candidate photoacceptor mediating PBM effects, other endogenous chromophores may also contribute to the biological response. Visual pigments and their retinoid chromophores absorb within portions of the visible spectrum that partially overlap with wavelengths used in PBM and could theoretically influence local photochemical events within photoreceptors. However, their specific contribution to PBM mechanisms in AMD remains uncertain, and it is currently unclear whether such interactions represent primary mediators of PBM effects or secondary modulators of mitochondrial signaling. At present, the available evidence supports a predominant role for mitochondrial signaling pathways, while additional chromophore-mediated effects warrant further investigation.

**Figure 3:**
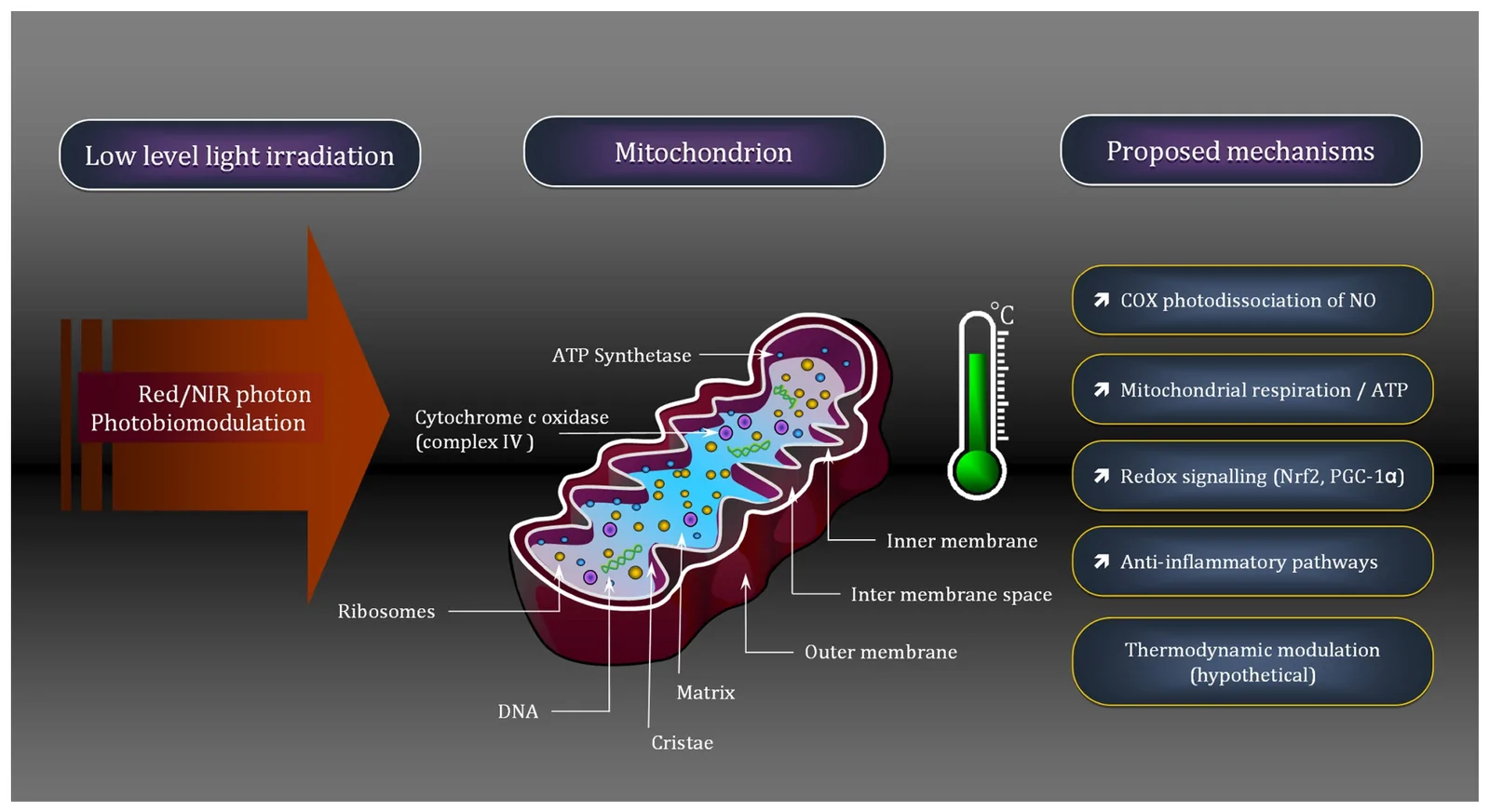
Mitochondrial respiratory complexes and proposed mechanisms of photobiomodulation. Schematic representation of the mitochondrial respiratory chain and the principal mechanisms proposed to mediate the biological effects of photobiomodulation (PBM). Red to near-infrared light may interact with respiratory chain components, particularly cytochrome c oxidase (Complex IV), leading to modulation of electron transport, nitric oxide photodissociation, and redox signaling pathways. These mechanisms may influence mitochondrial bioenergetics and cellular stress responses. Thermodynamic aspects of mitochondrial respiration are also indicated, although their physiological significance remains debated.

Beyond these core mitochondrial mechanisms, PBM has been proposed to influence a range of cellular processes, including modulation of oxidative stress, inflammatory signaling pathways, and mitochondrial biogenesis (Figure 3) [19]. While these mechanisms are often presented as complementary, they remain difficult to integrate into a coherent framework linking light–tissue interactions to the variability of metabolic and clinical responses observed in retinal disease.

Because many of these processes ultimately converge on mitochondrial respiration and cellular bioenergetics, understanding how PBM interacts with mitochondrial physiology is critical for clarifying its biological effects. In this context, recent studies examining the thermodynamic properties of mitochondrial respiration have introduced additional perspectives on how light–mitochondria interactions may influence cellular metabolism, further emphasizing the need for an integrated conceptual framework.

## 5. Mitochondrial Thermodynamics and the Thermal Microenvironment Hypothesis

Mitochondrial respiration is intrinsically associated with heat production. During oxidative phosphorylation, the electron transport chain converts chemical energy into a proton gradient that drives ATP synthesis, while a fraction of the free energy is dissipated as heat due to thermodynamic inefficiency [41,42,43,44]. As a result, mitochondrial activity is directly linked to local energy dissipation, with increases in respiratory flux expected to enhance heat production.

Recent experimental studies have suggested that actively respiring mitochondria may operate within local thermal microenvironments. Using a mitochondria-targeted thermosensitive probe, Chrétien and colleagues reported that mitochondria in metabolically active cells could exhibit temperatures higher than the surrounding cytosol under conditions of high respiratory activity [20]. These observations have raised the possibility that mitochondrial bioenergetic processes may be influenced by local physicochemical conditions within the organelle.

However, the magnitude and physiological significance of such temperature gradients remain debated. Several methodological limitations have been highlighted, particularly regarding fluorescence-based intracellular thermometry, which may be influenced by probe localization, chemical environment, and optical artifacts [22,45]. In addition, theoretical analyses of heat diffusion at the nanoscale suggest that maintaining large and stable temperature gradients within the intracellular environment may be unlikely [23].

**Figure 4:**
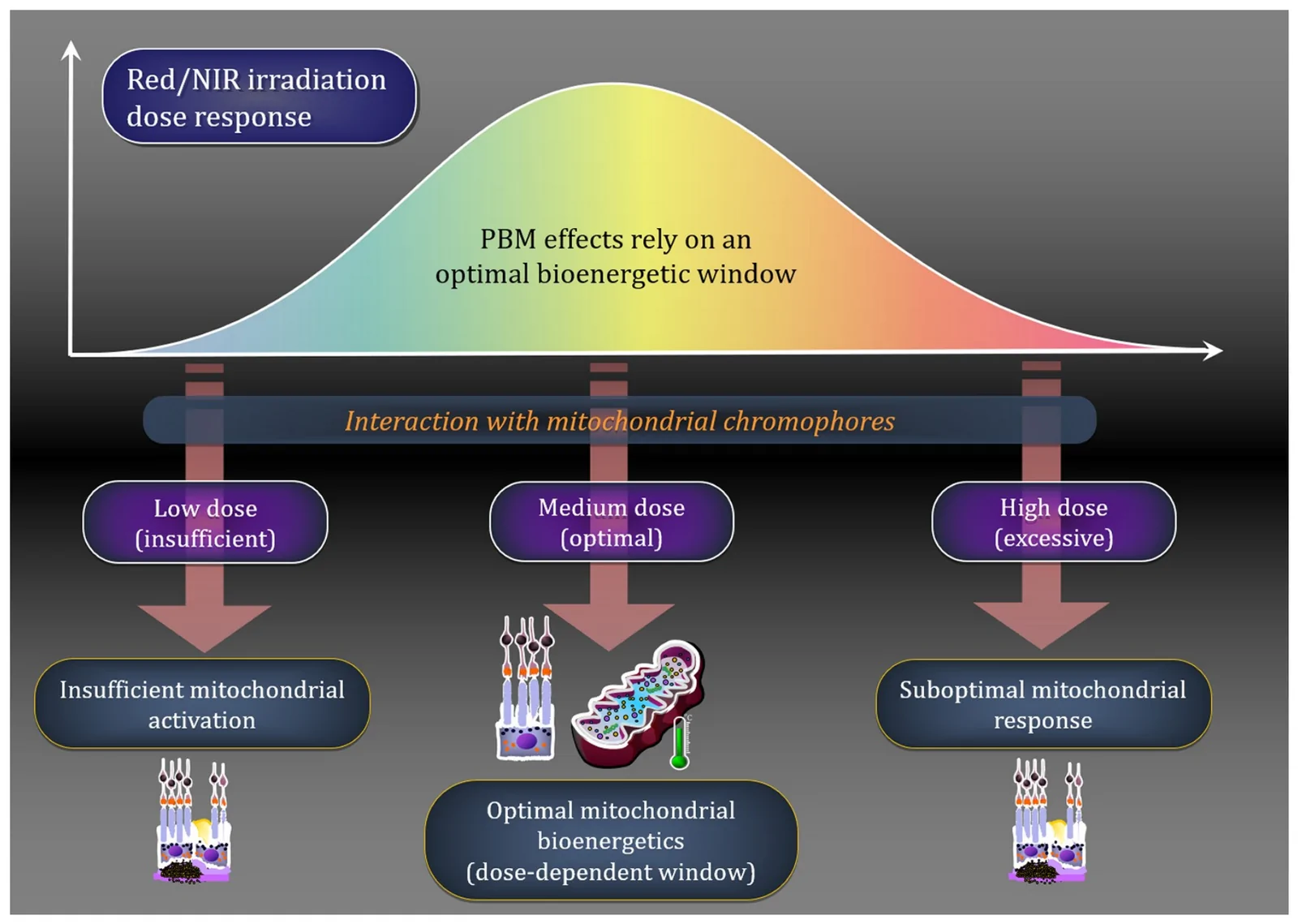
Dose-dependent bioenergetic window of photobiomodulation in retinal cells. Conceptual model illustrating the proposed dose-dependent bioenergetic window of photobiomodulation (PBM). Biological responses are limited at low levels of stimulation, maximal within an intermediate therapeutic range, and reduced at higher levels of stimulation. This biphasic relationship provides a framework for understanding variability in PBM responses. Adapted from conceptual models of PBM dose–response relationships described in neural systems [48]. Abbreviations: Red/NIR, red to near-infrared; RPE, retinal pigment epithelium.

In this context, the concept of mitochondrial thermal microenvironments should be interpreted cautiously. Rather than reflecting measurable bulk temperature differences between mitochondria and cytosol, it may correspond to subtle, spatially restricted physicochemical variations occurring at the level of the inner mitochondrial membrane. These variations could influence membrane properties, enzyme kinetics, and protein–protein interactions within respiratory supercomplexes, all of which are temperature-dependent processes (Figure 4).

Although the RPE and photoreceptor complex are emphasized because of their central role in AMD pathogenesis, mitochondrial populations are distributed throughout multiple retinal layers, including the plexiform and ganglion cell layers. PBM effects are therefore unlikely to be restricted to a single retinal compartment and may instead reflect broader modulation of retinal bioenergetics, although the RPE–photoreceptor complex remains particularly relevant because of its central role in AMD pathogenesis [32,46,47].

From a functional perspective, mitochondrial heat production reflects the balance between energy conversion and dissipation within the respiratory chain. Even modest changes in respiratory flux may therefore alter the local microenvironment of the inner mitochondrial membrane, potentially influencing oxidative phosphorylation efficiency, although direct experimental confirmation of this mechanism remains limited.

Within this framework, photobiomodulation may represent an external factor capable of modulating mitochondrial bioenergetics through its effects on respiratory chain activity. If light irradiation enhances electron transport, even small increases in metabolic flux could lead to subtle changes in local energy dissipation and microenvironmental conditions, which could in turn influence mitochondrial function. At present, this possibility remains a mechanistic hypothesis that requires direct experimental validation. This perspective integrates thermodynamic considerations into a broader bioenergetic framework and provides a mechanistic basis for the existence of a dose-dependent response to PBM.

## 6. A Bioenergetic Framework for Photobiomodulation in AMD

The potential relevance of photobiomodulation (PBM) for age-related macular degeneration is supported by the central role of mitochondrial metabolism in retinal physiology. Recent three-dimensional ultrastructural reconstructions of human RPE have demonstrated a dense basal mitochondrial layer adjacent to the choriocapillaris, emphasizing the remarkable metabolic specialization of the outer retina and reinforcing the relevance of mitochondria as potential targets of PBM interventions [32]. Photoreceptors and retinal pigment epithelium (RPE) cells operate under high metabolic demand and rely heavily on mitochondrial oxidative phosphorylation to sustain retinal function [9,30]. Mitochondrial alterations have been described in the RPE of patients with AMD, including structural abnormalities, mitochondrial DNA damage, and impaired respiratory chain activity [11,33]. Taken together, these observations support the view that mitochondrial dysfunction contributes to the metabolic vulnerability of the aging retina.

In this context, PBM has been proposed as a potential strategy to influence retinal bioenergetics. Experimental studies suggest that low-level light irradiation can modulate mitochondrial activity, including cytochrome c oxidase function and downstream redox signaling pathways [15,16]. Through these mechanisms, PBM has been proposed to enhance mitochondrial respiration, increase ATP production, and modulate oxidative stress responses, potentially improving cellular resilience in metabolically stressed retinal tissues.

Several clinical studies have explored the potential benefits of PBM in patients with AMD. Pilot studies have reported improvements in visual function and reductions in drusen volume, suggesting a possible effect on retinal metabolism and homeostasis [12,13]. However, the magnitude and reproducibility of these findings remain uncertain. Systematic reviews and meta-analyses have therefore remained cautious regarding the clinical efficacy of PBM, as heterogeneity in treatment protocols, patient populations, and outcome measures complicates interpretation of the available evidence [17,18]. In addition, the optimal dosimetry of PBM—including wavelength, irradiance, treatment duration, and treatment frequency—remains incompletely defined. Similar dose–response relationships have been described in neural systems exposed to PBM [48].

Unlike anti-VEGF therapy for neovascular AMD or complement inhibition in geographic atrophy, PBM has primarily been evaluated in earlier stages of AMD and aims to modulate retinal metabolism rather than directly target neovascularization or complement activation. Direct comparisons between these approaches remain difficult because of differences in patient populations, therapeutic objectives, endpoints, and follow-up durations.

Importantly, clinical responses to PBM appear to vary according to disease stage. Most recent studies have focused on patients with intermediate AMD, in whom functional improvements have been reported, notably in trials such as LIGHTSITE III [14]. In contrast, earlier studies including broader populations of “dry AMD” patients, encompassing more advanced stages such as geographic atrophy, have reported more variable and less consistent outcomes [13,25,49,50]. This heterogeneity may reflect differences in mitochondrial functional reserve and the extent of cellular loss, which are likely to be more pronounced in advanced disease.

**Figure 5:**
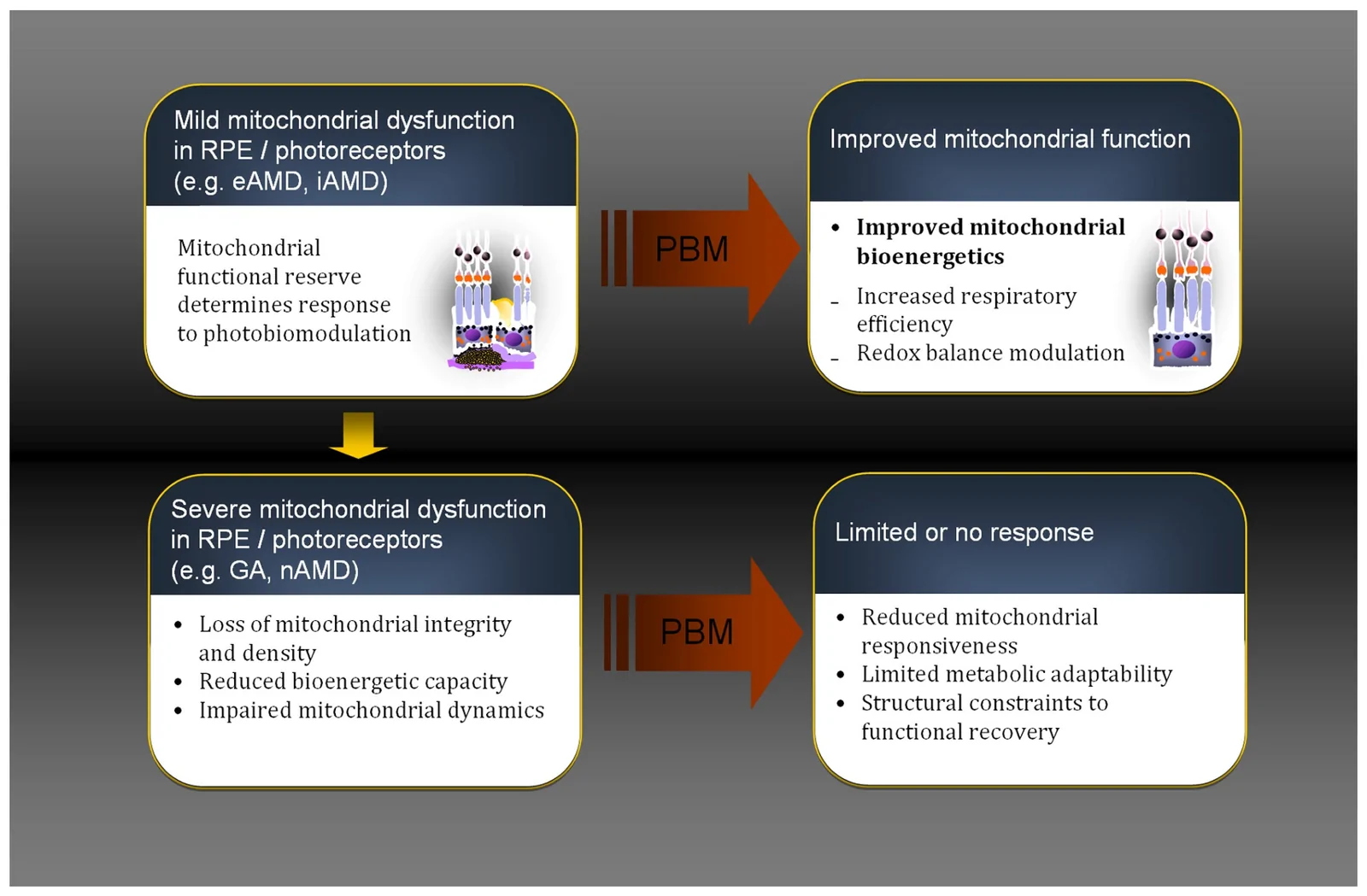
Stage-dependent response to photobiomodulation in retinal cells. Conceptual model illustrating the proposed relationship between disease stage, mitochondrial functional reserve, and response to photobiomodulation (PBM). Responsiveness is expected to decline progressively as mitochondrial dysfunction and irreversible cellular loss advance, suggesting greater responsiveness in earlier stages of AMD. This stage-dependent relationship complements the dose-dependent bioenergetic window illustrated in Figure 4. Abbreviations: eAMD, early age-related macular degeneration; iAMD, intermediate age-related macular degeneration; GA, geographic atrophy; nAMD, neovascular age-related macular degeneration; PBM, photobiomodulation; RPE, retinal pigment epithelium.

From a mechanistic perspective, these observations suggest that PBM effects depend on both the delivered light dose and the metabolic state of retinal tissues. In particular, the level of mitochondrial dysfunction may determine the capacity of retinal cells to respond to PBM, reflecting the concept of a variable mitochondrial functional reserve (Figure 5).

In early stages of AMD, characterized by mild to moderate mitochondrial dysfunction, retinal cells may retain sufficient bioenergetic reserve to respond to PBM, potentially resulting in improved mitochondrial bioenergetics and cellular function. Conversely, in advanced stages of retinal degeneration, where mitochondrial impairment is more severe and substantial cellular loss has already occurred, the biological substrate available for metabolic rescue may become progressively limited. These differences in metabolic state may partly reflect distinct pathogenic pathways within the outer retina, as illustrated by the coexistence of drusen and subretinal drusenoid deposits, which are associated with distinct cellular and metabolic vulnerabilities that may influence mitochondrial functional reserve.

Although no direct clinical measure of mitochondrial functional reserve currently exists, several structural and functional biomarkers may provide indirect estimates of this reserve. Potential surrogate markers include ellipsoid zone integrity, preserved photoreceptor layer structure, hyperreflective foci, transdifferentiated RPE cells, drusen phenotype, subretinal drusenoid deposits, retinal sensitivity, and functional measures such as dark adaptation. Hyperreflective foci and transdifferentiated RPE cells have been increasingly recognized as dynamic indicators of RPE phenotypic change and outer retinal remodeling in AMD [38]. Future metabolic imaging techniques may offer more direct assessments of retinal bioenergetic status. In addition, age, disease duration, genetic background, and retinal structural damage may act as confounding factors when interpreting these biomarkers.

Taken together, these observations support a model in which PBM responses are governed by two interdependent factors: a dose-dependent bioenergetic window and the underlying mitochondrial functional reserve of retinal cells. Within this framework, PBM may exert beneficial effects when delivered within an optimal bioenergetic window and in cellular contexts where mitochondrial dysfunction remains at least partially reversible (Figure 5).

## 7. Testable Implications

The conceptual framework proposed here leads to several testable predictions that can be evaluated in future experimental and clinical studies.

First, experimental studies in retinal and neural systems have suggested the existence of a biphasic response to PBM, with beneficial effects occurring within a limited range of stimulation. Consistent with this observation, PBM efficacy is expected to follow a dose-dependent bioenergetic window, in which both insufficient and excessive light exposure result in suboptimal responses, whereas an intermediate range of stimulation maximizes bioenergetic effects.

Second, recent clinical studies have reported more consistent functional improvements in patients with early and intermediate AMD than in advanced disease. Within the present framework, therapeutic responses are therefore expected to depend on the metabolic state of retinal tissues, with greater efficacy anticipated in eyes retaining sufficient mitochondrial functional reserve. This stage-dependent response predicts variable outcomes for identical PBM protocols across different AMD populations.

Third, increasing evidence suggests that structural and functional retinal biomarkers reflect disease severity and cellular resilience in AMD. Biomarkers such as ellipsoid zone integrity, hyperreflective foci, transdifferentiated RPE cells, subretinal drusenoid deposits, and dark adaptation abnormalities may therefore help identify patients most likely to benefit from PBM and monitor treatment response over time. Within this framework, these markers could serve as indirect indicators of retinal bioenergetic status and mitochondrial functional reserve.

Fourth, heterogeneity among clinical studies suggests that treatment parameters, including wavelength, irradiance, treatment duration, and treatment frequency, may substantially influence therapeutic outcomes. PBM protocols should therefore be optimized to maintain stimulation within an effective bioenergetic window while accounting for inter-individual variability in retinal metabolic status.

A conceptual summary of the potential clinical translation of this framework is provided in Table 3.

Finally, longitudinal studies integrating multimodal retinal imaging, functional outcomes, and emerging metabolic biomarkers are needed to establish whether PBM-induced bioenergetic modulation correlates with clinical responses and disease progression. Such studies could help validate the proposed relationship between mitochondrial functional reserve, retinal bioenergetics, and treatment efficacy.

Together, these predictions support a model in which PBM responses emerge from the interaction between a dose-dependent bioenergetic window and the mitochondrial functional reserve of retinal cells.

Despite encouraging results from the LIGHTSITE program, several limitations should be acknowledged. Most positive evidence currently derives from industry-sponsored trials using a single proprietary platform [12,13,14]. Furthermore, substantial heterogeneity exists across studies regarding patient selection, AMD phenotypes, treatment parameters, and outcome measures. Independent investigations such as the PBM4AMD study have confirmed short-term functional improvements but suggested that benefits may diminish over time [26]. Recent meta-analyses have therefore concluded that current evidence remains insufficient to establish definitive clinical efficacy [17,18]. These discrepancies may reflect both methodological heterogeneity and the existence of responder subgroups, emphasizing the need for biomarker-driven patient selection and longer independent randomized trials.

A simplified mathematical formulation illustrating the interaction between mitochondrial functional reserve and PBM dose-response is provided in the Mathematical Supplementary Materials available online.

## 8. Conclusions

Photobiomodulation has attracted growing interest as a potential non-invasive approach to modulate retinal metabolism in age-related macular degeneration. This rationale arises from the high energetic demands of the retina and the central role of mitochondrial oxidative phosphorylation in maintaining photoreceptor and retinal pigment epithelium function. Experimental studies suggest that low-level light irradiation may influence mitochondrial activity through multiple mechanisms, including modulation of cytochrome c oxidase, redox signaling pathways, and cellular stress responses.

Despite an expanding body of experimental and clinical literature, the biological effects of PBM remain incompletely understood. The diversity of proposed mechanisms reflects both the complexity of mitochondrial physiology and the heterogeneity of experimental models. In particular, thermodynamic considerations suggest that local mitochondrial microenvironments may influence bioenergetic processes, although the magnitude and physiological relevance of such effects remain debated.

From a clinical perspective, PBM represents a promising but still exploratory metabolic intervention in the early stages of AMD. Although encouraging functional signals have been reported, current evidence remains insufficient to establish definitive clinical efficacy or confirmed disease-modifying effects. Variability in treatment protocols, patient populations, and outcome measures continues to limit definitive conclusions regarding its efficacy and suggests that treatment response depends on both light parameters and the metabolic state of retinal tissues.

**Figure 6:**
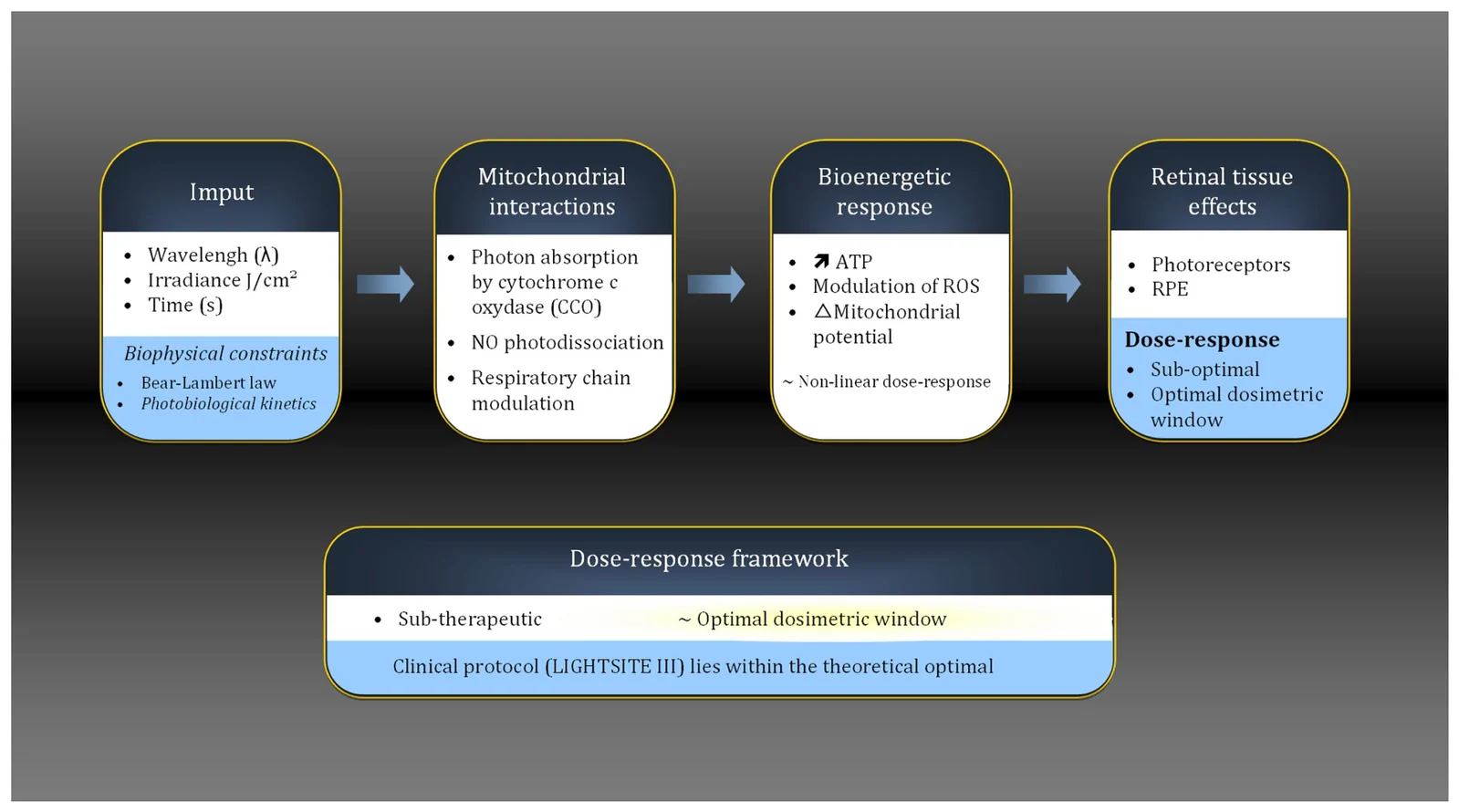
Photobiomodulation biophysical model (simplified). Conceptual summary of the framework proposed in this review. Photobiomodulation (PBM) is hypothesized to influence retinal bioenergetics through modulation of mitochondrial respiratory activity. The biological response is proposed to depend on the interaction between a dose-dependent bioenergetic window and the mitochondrial functional reserve of retinal cells. This framework may explain variability in PBM responses across AMD stages and clinical studies. Biophysical constraints governing light propagation and retinal energy absorption are incorporated into the model. Full mathematical derivations are provided in the Mathematical Supplementary Materials [20,51,52,53]. Abbreviations: PBM, photobiomodulation; ATP, adenosine triphosphate; ROS, reactive oxygen species.

Within this context, the framework proposed here links PBM effects to the interaction between a dose-dependent bioenergetic window and the mitochondrial functional reserve of retinal cells. Within this framework, therapeutic effects would be expected to occur preferentially when PBM is delivered within an optimal range of stimulation and in retinal tissues where mitochondrial dysfunction remains at least partially reversible, as summarized in the integrated framework shown in Figure 6.

Future research should aim to integrate advances in retinal imaging, mitochondrial biology, and photomedicine to better define both the cellular targets and optimal treatment parameters of PBM. Improved characterization of early AMD phenotypes, including intermediate AMD and subretinal drusenoid deposits, may further help identify patient populations most likely to benefit from such metabolic interventions.

## Figures and Tables

**Table 3:** Clinical translation of the proposed bioenergetic framework. Conceptual relationship between disease stage, mitochondrial functional reserve, and expected response to photobiomodulation (PBM). The table illustrates how differences in baseline metabolic status may influence responsiveness to PBM without implying specific treatment recommendations.

Disease Stage	Mitochondrial Functional Reserve	PBM Response	Expected Outcome
Early AMD	Mild dysfunction	High responsiveness	Improved bioenergetics and cellular function
Intermediate AMD	Moderate dysfunction	Variable or moderate responsiveness	Partial functional improvement
Advanced AMD (GA)	Severe dysfunction	Low or absent responsiveness	Minimal or absent functional effect

## Data Availability

Data presented in this study are available from the corresponding author upon request.

## Supplementary Materials

The following supporting information can be downloaded at: https://www.mdpi.com/article/10.3390/life16071098/s1.
