# Supplementary material for: Photobiomodulation in Age-Related Macular Degeneration: A Mitochondrial Bioenergetic Framework and Translational Perspective
Source: Life (Basel). 2026 Jun 30;16(7):1098. doi: 10.3390/life16071098 (PMC13412990; doi:10.3390/life16071098)
Supplement: Supplementary file 1 [file life-16-01098-s001.zip › life-4339759-supplementary.pdf]

Mathematical Supplementary Materials available online

THERMODYNAMIC CONSIDERATIONS UNDERLYING THE BIPHASIC RESPONSE TO PHOTOBIOMODULATION

Supplementary material associated with the manuscript:

" *Photobiomodulation in Age-Related Macular Degeneration: A Mitochondrial Bioenergetic Framework and Translational Perspective*"

Thomas Desmettre, MD, PhD; Serge Mordon, PhD

1. Introduction

Photobiomodulation (PBM) is characterized by a biphasic dose-response relationship, often referred to as the Arndt–Schulz law, in which insufficient stimulation produces little biological effect, moderate stimulation enhances cellular function, and excessive stimulation becomes ineffective or even detrimental. Although this phenomenon has been repeatedly documented experimentally, its mechanistic basis remains incompletely understood (1, 2).

In the present work, we propose that this behavior may emerge naturally from the interaction between two opposing thermodynamic processes occurring within mitochondria:

1. an increase in mitochondrial efficiency as temperature approaches an optimal operating point, and
2. a progressive increase in entropy production associated with excessive thermal excitation.

This document provides a simplified mathematical framework illustrating how these two effects can generate a bell-shaped biological response.

2. Mitochondrial Efficiency as a Function of Temperature

Experimental studies suggest that mitochondrial respiratory complexes do not operate with constant efficiency across temperatures. Instead, enzymatic activity increases with temperature until reaching an optimum beyond which function deteriorates.

Let

$$T$$

denote the local mitochondrial temperature and

$$T_{\text{opt}}$$

the temperature corresponding to maximal respiratory efficiency.

A simple quadratic approximation around this optimum can be written:

$$E(T) = E_{\max} - a\left(T - T_{\text{opt}}\right)^2,$$

where

- $E(T)$ is mitochondrial energetic efficiency,
- $E_{\max}$ is maximal efficiency,
- $a > 0$ determines the steepness of the decline.

This approximation corresponds to the first non-trivial term of a Taylor expansion around the optimum:

$$E(T) \approx E(T_{\text{opt}}) + \frac{1}{2}E''(T_{\text{opt}})(T - T_{\text{opt}})^2.$$

Because the optimum is a maximum,

$$E''(T_{\text{opt}}) < 0,$$

yielding the quadratic form above.

The model predicts that efficiency improves as temperature approaches

$$T_{\text{opt}},$$

but decreases once this optimum is exceeded.

Experimental measurements by Chretien and colleagues (3) have suggested that mitochondria may transiently operate near temperatures approaching approximately 50°C under physiological conditions, although this remains debated.

3. Entropy Production During Mitochondrial Activation

Biological systems operate far from thermodynamic equilibrium.

According to nonequilibrium thermodynamics, any irreversible process generates entropy (4). Increased electron transport activity therefore entails entropy production.

The local entropy production rate may be expressed as

$$\sigma = \sum_i J_i X_i,$$

where

- J_i denotes a generalized flux,
- X_i denotes the associated thermodynamic force.

For simplicity, we assume that overall entropy production increases monotonically with mitochondrial temperature and may be approximated by

$$S(T) = S_0 + b(T - T_0)^2,$$

where

- $S(T)$ represents entropy generation,
- S_0 is basal entropy production,
- T_0 is a reference temperature,
- $b > 0$.

This phenomenological relationship reflects the notion that increasingly intense metabolic activity incurs progressively larger thermodynamic costs.

4. Net Biological Benefit

The biological response to PBM is unlikely to depend solely on energetic efficiency.

Instead, the net effect may reflect a balance between useful work generated by mitochondria and the thermodynamic burden associated with maintaining that activity.

We therefore define a simplified benefit function:

$$B(T) = E(T) - \lambda S(T),$$

where

- $B(T)$ represents the overall biological benefit,
- λ is a weighting coefficient expressing the relative impact of entropy production.

Substituting the previous expressions yields

$$B(T) = E_{\text{max}} - a(T - T_{\text{opt}})^2 - \lambda[S_0 + b(T - T_0)^2].$$

If

$$T_0 \simeq T_{\text{opt}},$$

this simplifies to

$$B(T) = B_{\text{max}} - (a + \lambda b)(T - T_{\text{opt}})^2,$$

where

$$B_{\text{max}} = E_{\text{max}} - \lambda S_0.$$

Thus, the biological benefit exhibits a maximum near the optimal mitochondrial temperature.

5. Linking Temperature to Photonic Stimulation

The preceding formulation relates benefit to temperature. PBM, however, is controlled experimentally through light exposure.

Assuming that modest photonic stimulation induces small increases in mitochondrial temperature,

$$\Delta T = kD,$$

where

- D is the effective PBM dose,
- k is a proportionality constant.

Then

$$T = T_{\text{base}} + kD.$$

Substituting into the benefit function gives

$$B(D) = B_{\text{max}} - (a + \lambda b)(T_{\text{base}} + kD - T_{\text{opt}})^2.$$

The maximum response occurs when

$$D = D_{\text{opt}} = \frac{T_{\text{opt}} - T_{\text{base}}}{k}.$$

Consequently,

- low doses fail to sufficiently stimulate mitochondrial activity,
- intermediate doses maximize benefit,
- excessive doses move the system away from the optimum.

This naturally generates a biphasic dose-response relationship.

6. Relation to the Arndt–Schulz Law

The resulting curve is consistent with the experimentally observed Arndt–Schulz phenomenon.

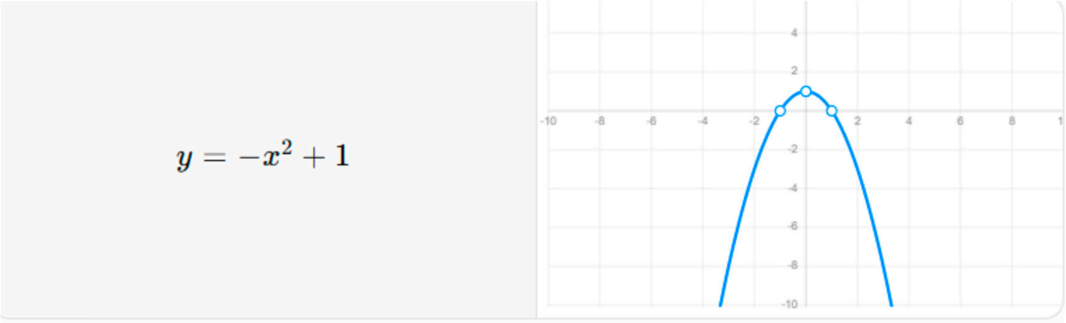

If dose is represented schematically by

$$x = D - D_{\mathrm{opt}},$$

the response becomes

$$R(D) = R_{\mathrm{max}} - \beta(D - D_{\mathrm{opt}})^2,$$

with

$$\beta > 0.$$

The graph displays the characteristic inverted-U profile:

- negligible effect at low doses,
- optimal stimulation at intermediate doses,
- declining efficacy beyond the optimum.

This simple formulation captures the essential qualitative features repeatedly described in PBM experiments.

7. Biological Interpretation

Several non-mutually exclusive mechanisms may contribute to this thermodynamic optimum:

- enhanced cytochrome c oxidase activity near optimal conditions;
- reversible nitric oxide photodissociation improving electron transport;
- transient increases in ATP synthesis;
- activation of redox-sensitive signaling pathways;
- increased reactive oxygen species generation at excessive stimulation levels;
- growing energetic costs associated with maintaining homeostasis.

Within this framework, PBM does not continuously improve mitochondrial performance. Instead, it shifts mitochondria toward a restricted operational window where energetic gain temporarily exceeds thermodynamic cost.

8. Limitations of the Model

This mathematical framework is intentionally simplified and several assumptions should be emphasized.

First, the existence and magnitude of mitochondrial temperature gradients remain debated.

Second, the relationship between PBM dose and temperature increase is unlikely to be strictly linear.

Third, entropy production was represented using a phenomenological approximation rather than derived from detailed biochemical kinetics.

Fourth, multiple PBM mechanisms independent of temperature, including direct photochemical signaling pathways, may also contribute substantially to the observed biological response.

Accordingly, the present model should not be interpreted as a quantitative predictive model, but rather as a conceptual thermodynamic explanation illustrating how an optimal biological response may emerge from the balance between increasing mitochondrial efficiency and increasing entropy production.

9. Conclusion

The biphasic response to photobiomodulation can be interpreted as the consequence of two competing processes operating within mitochondria: the enhancement of energetic efficiency as the system approaches an optimal operating state and the concomitant rise in entropy production accompanying increasing metabolic activation.

Despite its simplicity, this framework reproduces the principal qualitative feature of PBM—the existence of an optimal therapeutic window—and provides a thermodynamically grounded perspective that may help integrate diverse experimental observations into a unified conceptual model.

References cited in this supplementary document

1. Hamblin MR. Mechanisms and applications of the anti-inflammatory effects of photobiomodulation. *AIMS Biophysics*. 2017;4:337–361.
2. Hennessy, M, Hamblin, M. R. Photobiomodulation and the brain: a new paradigm. J Opt. 2017, 19(1):013003.
3. Chretien D, Bénit P, Ha HH, et al. Mitochondria are physiologically maintained at close to 50°C. *PLoS Biology*. 2018;16:e2003992.
4. Prigogine I. *Introduction to Thermodynamics of Irreversible Processes*. 3rd ed. New York: Interscience; 1967.
